# Study on the effects of nitrilotriproprionic acid and 4,5-dihydroxy-1,3-benzene disulphonate on the fractionation of beryllium in human serum using graphite furnace atomic absorption spectrometry

**DOI:** 10.1186/1752-153X-2-10

**Published:** 2008-05-14

**Authors:** Chadi H Stephan, Michel Fournier, Pauline Brousseau, Sébastien Sauvé

**Affiliations:** 1 Department of Chemistry, Université de Montréal, PO 6128, Station Centre-Ville, Montréal, QC, H3C 3J7, Canada; 2 INRS-Institut Armand-Frappier, 245 Hymus, Pointe-Claire, QC, H9R 3G6, Canada

## Abstract

**Background:**

Occupational exposure to beryllium may cause Chronic Beryllium Disease (CBD), a lung disorder initiated by an electrostatic interaction with the MHC class II human leukocyte antigen (HLA). Molecular studies have found a significant correlation between the electrostatic potential at the HLA-DP surface and disease susceptibility. CBD can therefore be treated by chelation therapy. In this work, we studied the effect of two complexing agents, nitrilotriproprionic acid (NTP) and 4,5-dihydroxy-1,3-benzene disulphonate (Tiron), on the fractionation of beryllium in human serum analysed by graphite furnace atomic absorption spectrometry (GFAAS).

**Results:**

We found the average serum beryllium concentration of fourteen non-exposed individuals to be 0.53 (± 0.14) μg l^-1^, with 21 (± 3)% of the beryllium mass bound to the low molecular weight fraction (LMW), and 79 (± 3)% bound to the high molecular weight fraction (HMW). The addition of Tiron increased the beryllium mass in the HMW fraction, while NTP was not seen to have any influence on the fractionation of beryllium between the two fractions. NTP was, however, shown to complex 94.5% of the Be mass in the LMW fraction. The beryllium GFAAS detection limit, calculated as three times the standard deviation of 10 replicates of the lowest standard (0.05 μg L^-1^), was 6.0 (± 0.2) ng L^-1^.

**Conclusion:**

The concentration of beryllium or its fractionation in human serum was not affected by sex or smoking habit. On average, three quarters of the beryllium in serum were found in the HMW fraction. Of the two ligands tested, only Tiron was effective in mobilising beryllium under physiological conditions, thus increasing the Be content in the HMW fraction.

## Background

Beryllium, the first of the alkaline earth metals, is naturally found in mineral rocks, coal, soil, and volcanic dust [1]. Beryllium ore is mined and purified for its use in nuclear reactors, weapons, aircraft and space vehicle structures, x-ray machines, as well as telecommunication and high-tech devices [2]. Occupational exposure to beryllium may cause Chronic Beryllium Disease (CBD), a lung disorder characterised by a granulomatous inflammation initiated by an electrostatic interaction with the MHC class II human leukocyte antigen (HLA) [3,4]. Molecular epidemiological studies have shown that interaction between beryllium and specific HLA-DP alleles is a factor in disease susceptibility [5]. Furthermore, molecular modelling has been used to investigate a potential mechanistic basis for these observations. A significant correlation has been found between the risk of chronic beryllium disease associated with specific alleles, and the predicted electrostatic surface potential, suggesting that the alleles associated with the most negatively charged proteins carry the greatest risk of beryllium sensitisation and disease [6].

**Figure 1:**
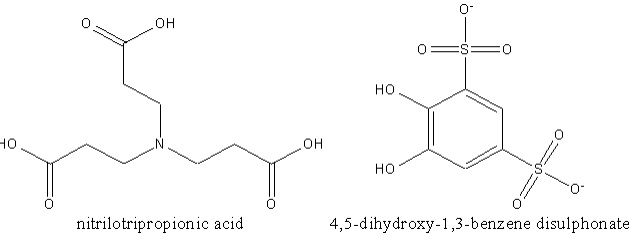
Chemical structures of nitrilotripropionic acid (NTP) and 4,5-dihydroxy-1,3-benzene disulphonate (Tiron).

At present, CBD can be treated but not cured. Adrenocortical steroids such as prednisone, prednisolone and dexamethasone act to reduce the inflammation and immune response to beryllium but cannot eliminate beryllium from organs or tissues, a key factor in stopping CBD [7]. Chelation therapy [1,8,9] or metal encapsulation [10] can eventually provide an alternative or adjunctive treatment to accelerate beryllium clearance from organs and tissues. In this work, we looked at the natural distribution of beryllium in serum and investigated the effect of two sequestering agents, nitrilotriproprionic acid (NTP) and 4,5-dihydroxy-1,3-benzene disulphonate (Tiron) (Figure 1), on the distribution of beryllium. We used a mobilisation index (MI) [11-13] to represent the relative ability of a complexing agent to compete for the metal of interest and mobilise it under physiological conditions. The MI is defined as:

$$MI=\frac{\left(\begin{array}{cc} total & \begin{array}{ccc} concentration & of & \begin{array}{ccc} low & molecular & \begin{array}{cc} weight & beryllium \end{array} \end{array} \end{array}\\ in & \begin{array}{ccc} the & presence & \begin{array}{ccc} of & the & \begin{array}{cc} complexing & agent \end{array} \end{array} \end{array} \end{array}\right)}{\left(\begin{array}{cc} total & \begin{array}{ccc} concentration & of & \begin{array}{ccc} low & molecular & \begin{array}{cc} weight & beryllium \end{array} \end{array} \end{array}\\ in & \begin{array}{ccc} the & absence & \begin{array}{ccc} of & the & \begin{array}{cc} complexing & agent \end{array} \end{array} \end{array} \end{array}\right)}$$

Nitrilotripropionic acid was selected because of its physico-chemical properties: it complexes beryllium in a tetrahedral complex [Be(NTP)]^- ^where the beryllium cation lies at the centre of a slightly distorted tetrahedron of C_3υ _symmetry, with a longer Be-N bond and three equal Be-O bonds [14]. Tiron is a hydrophilic chelator and was selected because of its reported efficiency in mobilising beryllium [15,16], restoring the altered biochemical parameters [17] and improving the altered hepatorenal biochemistry and ultramorphology in different rat tissues and organs [18].

Beryllium, like any other metal ions in serum, can be fractionated into four distinct groups: rigidly bound to metalloproteins; loosely bound to other types of proteins (labile equilibrium); complexed by the so-called low-molecular-weight fraction (LMW); and occurring as free (or hydrated) metal ions [12].

In this study, we looked first at the natural distribution of beryllium in serum and the influence of NTP and Tiron on this distribution. We focused on two operationally-defined fractions; the first fraction combines the Be rigidly and loosely bound to proteins, and is called the high molecular weight fraction (HMW); whilst the second fraction, that is the low molecular weight fraction (LMW), combines the Be complexed to the low molecular-weight fraction or that which occurs in its free ionic form. We separated the HMW and LMW fractions by ultrafiltration with Centricon centrifugal filter devices at a 10,000 nominal molecular weight limit (NMWL) cut-off. Beryllium quantification in serum and serum fractions was carried out using an optimised graphite furnace atomic absorption spectrometer (GF-AAS). The accuracy of the analytical method was tested using Seronorm samples (Seronorm trace elements whole blood level 2, STEWB; Ref # 201605; Lot # 0503109).

## Results and discussion

### Beryllium in serum

The average beryllium concentration in fourteen non-exposed individuals (9 females and 5 males; 9 non smokers and 5 smokers) found to be 0.53 (± 0.14) μg L^-1^. Neither sex nor smoking habit was shown to have a significant influence on the concentration of beryllium in serum (independent sample T-test, p > 0.05). The method detection limit, calculated as three times the standard deviation of 10 replicates of the lowest standard (0.05 μg L^-1^), was 6.0 (± 0.2) ng L^-1^. The accuracy of the method varied from 99 to 104% and was verified by analysing a control sample (STEWB level 2 certified blood material with a concentration of 5.9 (± 0.5) μg Be L^-1^).

### Beryllium distribution in serum

**Figure 2:**
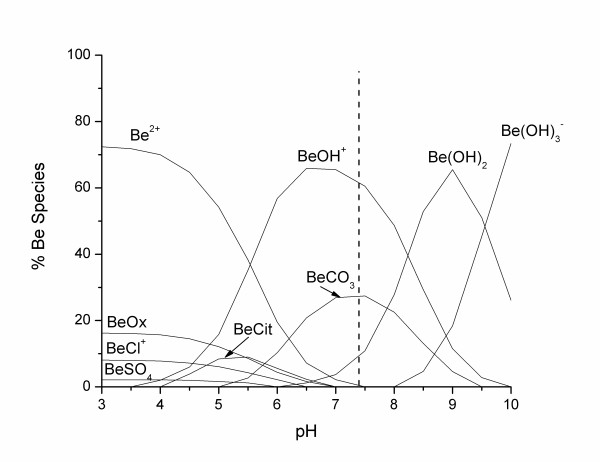
Speciation of Be in the LMW of serum fluid ([Be] 5.8*10^-5 ^mM; [Ca] 1.3 mM; [Cl] 108 mM; [CO_3_] 24 mM; [K] 4.2 mM; [Mg] 0.8 mM; [Na] 142 mM; [PO_4_] 2 mM; [SO_4_] 0.5 mM; [Cit] 0.1 mM; [Ox] 0.01 mM).

On average, 21 (± 3)% of the beryllium mass was bound to the LMW fraction, with 79 (± 3)% bound to the HMW fraction (Table 1). Neither sex nor smoking habit was shown to have a significant influence on the distribution of beryllium between the LMW and the HMW fractions (independent sample T-test, p > 0.05). These findings agree with the work of Stiefel *et al*. [19] conducted on human and guinea pig blood. They reported that the corpuscular part of all samples contained from 2 to 10% of the total Be, the LMW fraction contained between 17 and 33%, with 60 to 73% of the total Be bound to the HMW fraction – or more specifically bound to the pre-albumins and the γ-globulins fractions. They also reported that the Be distribution between the two protein regions depends on the absolute concentration of Be in blood. At concentrations lower than 1 μg L^-1^, most of the Be mass (> 90%) was bound to the γ-globulins fraction, while at higher concentrations most of the Be mass was found bound to the pre-albumins fraction. This is very similar to the chemical speciation of aluminium in human serum, were almost all the Al mass in the HMW fractions was bound to transferrin belonging to the γ-globulins protein fractions [20-22]. Beryllium and aluminium have very similar chemical properties [1]. In an attempt to better understand beryllium chemistry in human body fluids, Sutton and Burastero [23] have simulated the speciation of beryllium in plasma fluid (LMW fraction) and reported most of the beryllium at pH 7.4 to be aqueous BeCO_3 _(49.4%) and BeOH^+ ^(39.9%). A reproduction of this speciation is presented in Figure 2, where in using the chemical equilibrium software Mineql+ [24] we show that adding citrate and oxalate, should complex most of the Al in the LMW human serum [20,22,25-27]. We obtained similar results and found that most of the beryllium at pH 7.4 is shown to exist as aqueous Be(OH)^+ ^(60.5%), BeCO_3 _(27.4%) and Be(OH)_2 _(10.2%). Neither citrate nor oxalate influenced the speciation of beryllium at serum pH, although both ligands complexed beryllium at acidic pH with maxima of 16.2% (at pH = 3) and 9% (at pH = 5.5) for Be(oxalate) and Be(citrate) respectively.

**Table 1:** Concentration and natural distribution of beryllium in the human serum of non-exposed individuals.

Sample	Sex	Smoking	Serum (μg L^-1^)	LMW (%)	HMW (%)
1	F	S	0.54 (0.08)	22 (2)	78 (3)
2	F	NS	0.46 (0.04)	24 (3)	76 (3)
3	F	S	0.47 (0.09)	19 (1)	81 (1)
4	F	NS	0.42 (0.05)	24 (2)	76 (2)
5	F	NS	0.49 (0.07)	21 (2)	79 (2)
6	M	NS	0.44 (0.02)	25 (3)	75 (3)
7	F	NS	0.45 (0.01)	25 (3)	75 (3)
8	F	NS	0.44 (0.01)	19 (1)	81 (1)
9	F	NS	0.46 (0.01)	24 (3)	76 (3)
10	M	NS	0.45 (0.01)	16 (3)	84 (3)
11	M	S	0.78 (0.03)	23 (4)	77 (4)
12	F	NS	0.69 (0.03)	21 (5)	79 (5)
13	M	S	0.45 (0.04)	20 (3	80 (3)
14	M	S	0.86 (0.01)	21 (3)	79 (3)

Mean			0.53 (0.14)	21 (3)	79 (3)

F = female; M = male; S = smoking; NS = non smoking

In an attempt to investigate the chelation effect of Tiron and NTP, as well as study their effect on the repartition of beryllium in human serum, dose-response experiments were conducted showing the influence of both complexing agents on the repartition of beryllium between the two serum fractions. We tested different equilibration times (2, 4 and 6 h), but found no differences for both ligands, suggesting that the exchange reactions rates are almost instantaneous and that equilibrium is reached within the 2 hour equilibration timeframe employed. This is similar to the Al-transferrin exchange reaction rate with desferrioxamine B (DFO) where the addition of DFO increased the amount of ultrafiltrable Al sharply up to 90% of the total plasma Al content [28,29]. Due to the similarity between Be and Al "hard ions", and DFO, NTP and Tiron "complexing agents with soft oxygen donors", we should expect almost instant exchange reaction rates for the Be-Tiron-HMW and Be-NTP-HMW systems, thus confirming that the 2-h equilibration time is more than enough for the exchange to occur.

**Figure 3:**
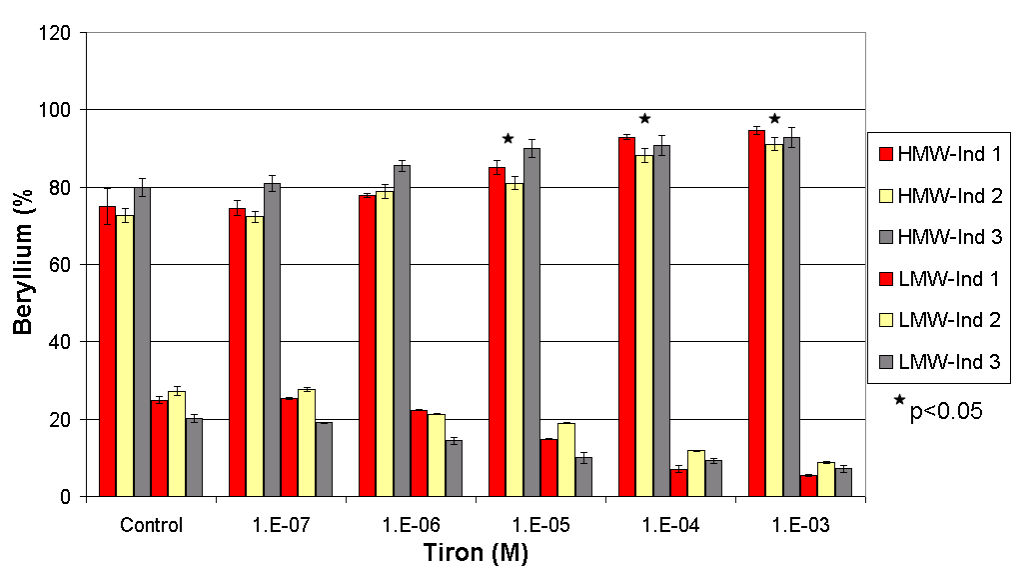
Influence of Tiron on the repartition of beryllium between the HMW and the LMW fractions in three spiked individuals. * Significant difference calculated by One-Way Anova, Fisher's LSD.

**Figure 4:**
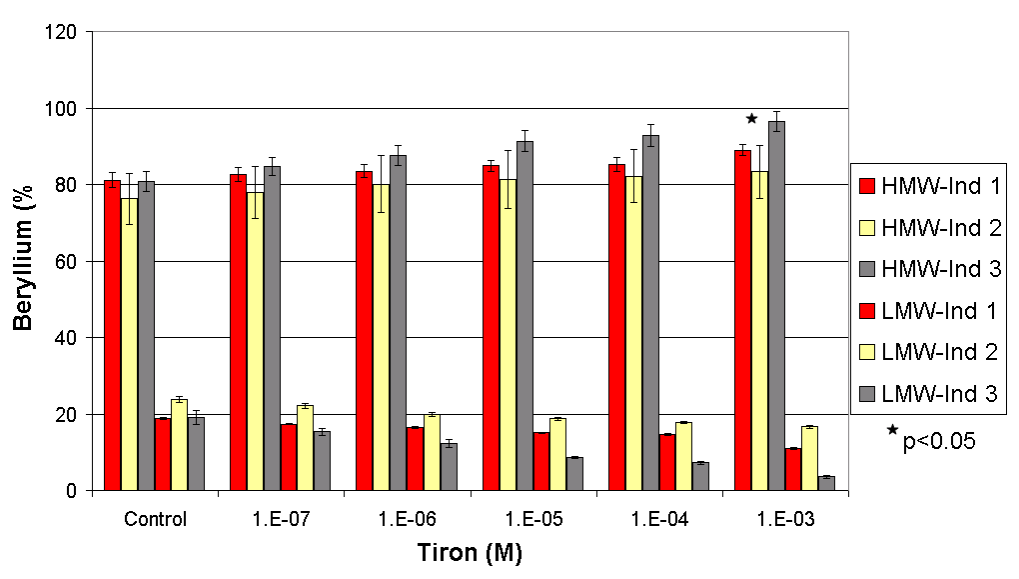
Influence of Tiron on the repartition of beryllium between the HMW and the LMW fractions in three non-spiked individuals. * Significant difference calculated by One-Way Anova, Fisher's LSD.

**Figure 5:**
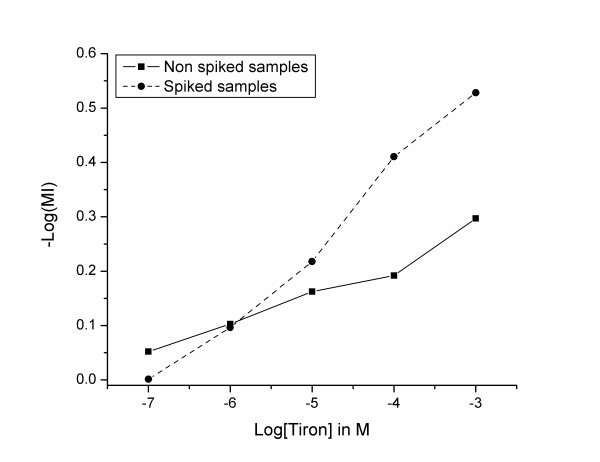
Mobilisation Index (MI) of Be as a function of Tiron concentration.

In spiked and non-spiked samples (Figures 3 and 4), Tiron showed an increasing pattern of the beryllium mass in the HMW fraction. Such results contradict the general behaviour of complexing agents, which usually tend to increase the metal concentration in the LMW fraction [12,13]. In the spiked samples, the addition of Tiron increased the mass fraction of beryllium in the HMW from an average of 76 (± 4)%, reaching a maximum of 93 (± 2)% at 10^-3 ^M. Conversely, the average concentration of beryllium in the LMW fraction decreased from 24 (± 4)% to 7 (± 2)%. This increase in the Be-HMW fraction becomes significant at 10^-5 ^M (One-Way Anova, Fisher's LSD, with P < 0.05). We observed the same pattern in non-spiked samples. The HMW beryllium content increased from an average of 79 (± 3)% to 90 (± 4)% at 10^-3 ^M, while the LMW beryllium content decreased from 20 (± 3)% to 10 (± 4)%. This change in the Be-HMW fraction is only significant at 10^-3 ^M. These observations confirm that Tiron complexes Be under serum conditions and increases the beryllium content in the HMW fraction by generating a mobilisation index that decreases with increasing Tiron concentrations (Figure 5). Tiron complexes Be in a 2:1 ratio [30,31], most probably generating a less charged complex that tends to be more soluble in the HMW fraction than in the hydrophilic LMW fraction. We suspect that such behaviour might increase the toxicity of beryllium, but several recent studies have shown that Tiron (with or without the aid of adjuvants) restored the altered biochemical physiological parameters and oxidative stress response in rats exposed to beryllium [15,18,31].

**Figure 6:**
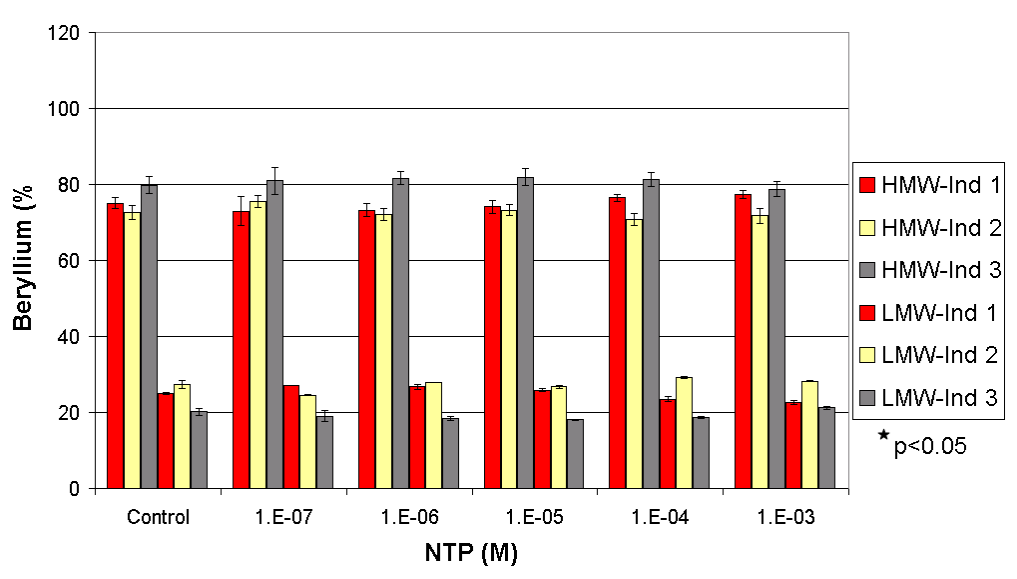
Influence of NTP on the repartition of beryllium between the HMW and the LMW fractions in three spiked individuals. * Significant difference calculated by One-Way Anova, Fisher's LSD.

**Figure 7:**
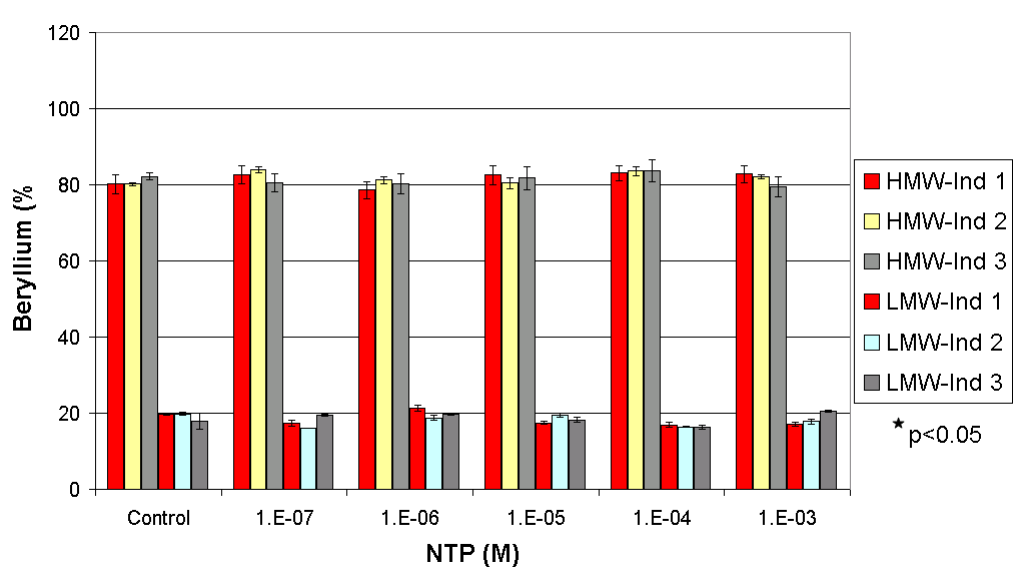
Influence of NTP on the repartition of beryllium between the HMW and the LMW fractions in three non-spiked individuals. * Significant difference calculated by One-Way Anova, Fisher's LSD.

**Figure 8:**
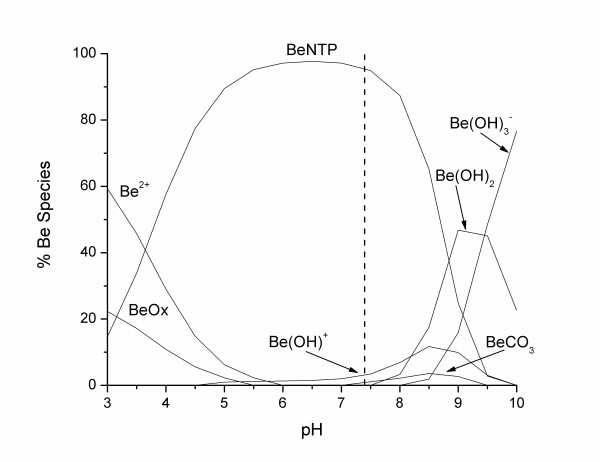
Influence of NTP on the speciation of Be in the LMW of serum fluid ([Be] 5.8*10^-5 ^mM; [NTP] 10^-4 ^mM; [Ca] 1.3 mM; [Cl] 108 mM; [CO_3_] 24 mM; [K] 4.2 mM; [Mg] 0.8 mM; [Na] 142 mM; [PO_4_] 2 mM; [SO_4_] 0.5 mM; [Cit] 0.1 mM; [Ox] 0.01 mM).

NTP did not show any significant influence on the beryllium partitioning either in spiked or non-spiked samples (Figures 6 and 7). In spiked samples, the HMW average beryllium fractionation did not change significantly (One-Way Anova, Fisher's LSD, with P < 0.05) with increasing NTP concentration. We obtained a stable pattern that varied from 76 (± 4)% for the control to 77 (± 5)% at 10^-3 ^M. Similar observations were observed in non-spiked samples. The HMW average beryllium content pattern varied from 77 (± 5)% for the control to 80 (± 4)% at 10^-3 ^M. Under physiological serum conditions, NTP did not influence the MW distribution of Be between the HMW and LMW fractions, but showed a capacity to complex most of the beryllium in the LMW, as shown by the chemical speciation simulations (Figure 8). At a concentration of 10^-7 ^M, NTP was shown to complex 94.5% of the Be in the LWM followed by 3.4% and 1.1% of the Be occurring as Be(OH)^+ ^and BeCO_3_, respectively. These results support earlier findings which suggest that NTP would be an ideal complexing agent for Be [14].

## Conclusion

Neither sex nor smoking habit was shown to influence beryllium concentration or its fractionation in human serum. On average, three quarters of the beryllium in serum were found to be bind to the HMW fraction. Tiron, unlike NTP, showed a significant interaction with beryllium under physiological conditions by increasing the Be content in the HMW fraction – in contrast to that which is usually observed for standard complexing agents used in chelation therapy. The addition of NTP did not affect the MW distribution between the two fractions but was shown to complex most of the Be in the LMW fraction. Further work could employ anion exchange fast protein liquid chromatography (FPLC) coupled with electrospray tandem mass spectrometry (ES-MS-MS) or inductively coupled plasma mass spectrometry to confirm the nature of the ligands or proteins that complex Be in both serum fractions [20-22,27].

## Experimental

### Reagents

All of the reagents used were of analytical grade or better. Antifoam B silicone emulsion (J.T. Baker, NJ, USA), ammonium hydroxide (certified A.C.S. Plus, Fisher scientific, NJ, USA), ethylenediaminetetraacetic acid disodium salt dihydrate (EDTA) (Fluka chemika, Switzerland), Triton X-100 (Acros, NJ, USA), nitric acid (trace metal grade, Fisher Scientific, Ontario, Canada), beryllium plasma standard solution (Specpure, Alfa Aesar, MA, USA), nitrilotripropionic acid (NTP) (MP Biomedicals, Ohio, USA) and 4,5-dihydroxy-1,3-benzene disulphonate (Tiron) (Acros Organics, New Jersey, USA).

### Solutions

Serum samples were diluted with a Nash reagent (NR) solution prepared weekly and containing 5% (v/v) nitric acid, 5% (v/v) of ammonium hydroxide, 0.2% (v/v) Triton X-100, 0.2% (v/v) antifoam B and 0.5% (w/v) of EDTA in distilled-deionised water. Solutions of NTP and Tiron were prepared at 10^-1^, 10^-2^, 10^-3^, 10^-4 ^and 10^-5 ^M by dissolving the appropriate mass in 2% (v/v) HNO_3 _aqueous solution. A 50 μg L^-1 ^working Be (II) solution was prepared by dilution of the beryllium stock solution (1000 μg L^-1^) in 2% (v/v) HNO_3_. The Be standard solution was prepared daily by dilution of the working Be (II) solution with the Nash reagent to give a final concentration of 0.5 μg L^-1^.

### Sample preparation

Blood from unexposed individuals was collected in BD vacutaine SST tubes (BD Franklin lakes, New Jersey, USA). Tubes were left at room temperature for 30 minutes to allow clot formation and then centrifuged at 500 g for 10 minutes. Supernatants of each individual were collected and recombined to ensure sample homogenisation. Each sample was divided into twelve 2.5 mL sub-samples in borosilicate disposable culture tubes, two served as controls, five were spiked with NTP and five were spiked with Tiron at concentrations ranging from 10^-7 ^to 10^-3 ^M. Sub-samples were left to equilibrate for two hours. A 2 mL aliquot of each sub-sample was transferred to a Centricon centrifugal device with ultracel YM-10 (10,000 MWCO) (Millipore Corporation, Ireland) and centrifuged at 4500 g for 90 minutes. The LMW and HMW fractions obtained were analysed for beryllium by graphite furnace atomic absorption spectrometry (GFAAS) after respective 2-fold and 5-fold dilutions with NR. The same experimental procedure was repeated on serum samples that were spiked with 1 μg L^-1 ^of beryllium. We also measured the beryllium concentration in fourteen individuals and looked at the natural distribution of beryllium between the HMW and LMW fractions. Serum was diluted 5-fold with NR before GFAAS analysis.

### Graphite furnace atomic absorption spectrometer

A Varian AA280Z Zeeman atomic absorption spectrometer, equipped with a Zeeman background correction, GTA 120 graphite tube atomiser and PSD 120 programmable sample dispenser was used for the atomic absorption measurement of beryllium at 234.9 nm with a spectral bandwidth of 1.0 nm. A beryllium hollow cathode lamp (Varian, Part No. 5610100500) was used as a light source operated at 5 mA. Pyrolytic graphite coated partitioned tubes (Varian partition tubes, Part No. 63-100012-00) were used for all experiments. High purity Argon (99.99%) was used as the carrier gas. A beryllium hollow cathode lamp (Varian, Part No. 5610100500) was used as a light source. Instrument control, sample results, signal graphics and data collection are controlled by the SpectrAA Worksheet software for the Windows^® ^XP operating system. Peak area values were used for beryllium signal and background (BG) measurements. The instrumental conditions and the furnace program are listed in Table 2.

**Table 2:** The furnace programme for the determination of beryllium in serum.

Step	Temp (°C)	Time (s)	Argon flow rate (L min^-1^)
Drying	85	5	0.3
Drying	95	30	0.3
Drying	120	20	0.3
Pre-pyrolysis	450	22	0.3
Pyrolysis	1000	17	0.3
Atomizing	2900	3	0
Cleaning	2900	2	0.3

### Chemical Speciation

For the chemical speciation calculations, we used MINEQL+ (version 4.5 for Windows – Environmental Research Software, Hallowell, ME), a chemical equilibrium modelling system that can be used to perform calculations at low temperatures (0–50°C) and low to moderate ionic strength (< 0.5 M). MINEQL+ operates over three steps: creation of a system by selecting chemical components from a menu with the possibility of adding new ligands, then scanning the thermodynamic database and finally running the calculations with actual measured concentrations included. The output data module yields the activity for each species of each component. Our chemical speciation calculations were made assuming the following parameters (unless specified otherwise): T = 37°C (fixed), ionic strength: I = 0.01 M (fixed), Log pCO_2 _= -3.5 (open atmosphere). Different chemical equilibrium software should yield similar results when the same stability constants are used. The following input data were added to the MINEQL+ thermodynamic database; Log K_Be-Ox _= 3.47, Log K_Be-(Ox)2 _= 5.24, Log K_Be-NTP _= 13.94, Log K_H3-NTP _= 11.54, Log K_H2-NTP _^-1 ^= 10.31 and Log K_H-NTP _^-2 ^= 7.59.

### Statistical analysis

Statistical analysis was performed with SPSS (version 13 for Windows, SPSS Inc, Chicago, IL) using independent sample T-tests to monitor significant differences among groups. We also used One-Way ANOVA tests with Fisher's LSD (least significant difference) to make pair wise comparisons as a way of monitoring significant changes within a group.

## Authors' contributions

All authors contributed to the experimental design. CHS carried out nearly all of the laboratory work, the initial interpretation of the data and the initial write-up. PB and MF contributed their expertise especially with the biological work, and SS focused on the analytical chemistry.
